# Supplementary material for: The interactome of intact mitochondria by cross-linking mass spectrometry provides evidence for coexisting respiratory supercomplexes
Source: Mol Cell Proteomics. 2017 Dec 8;17(2):216–32. doi: 10.1074/mcp.RA117.000470 (PMC5795388; doi:10.1074/mcp.RA117.000470)
Supplement: Supplemental Data [file supp_RA117.000470_133922_0_supp_23978_fzffwf.zip › spectra_annotation/mito_spectra_annotation/3-1-10-1-13-1.pdf]

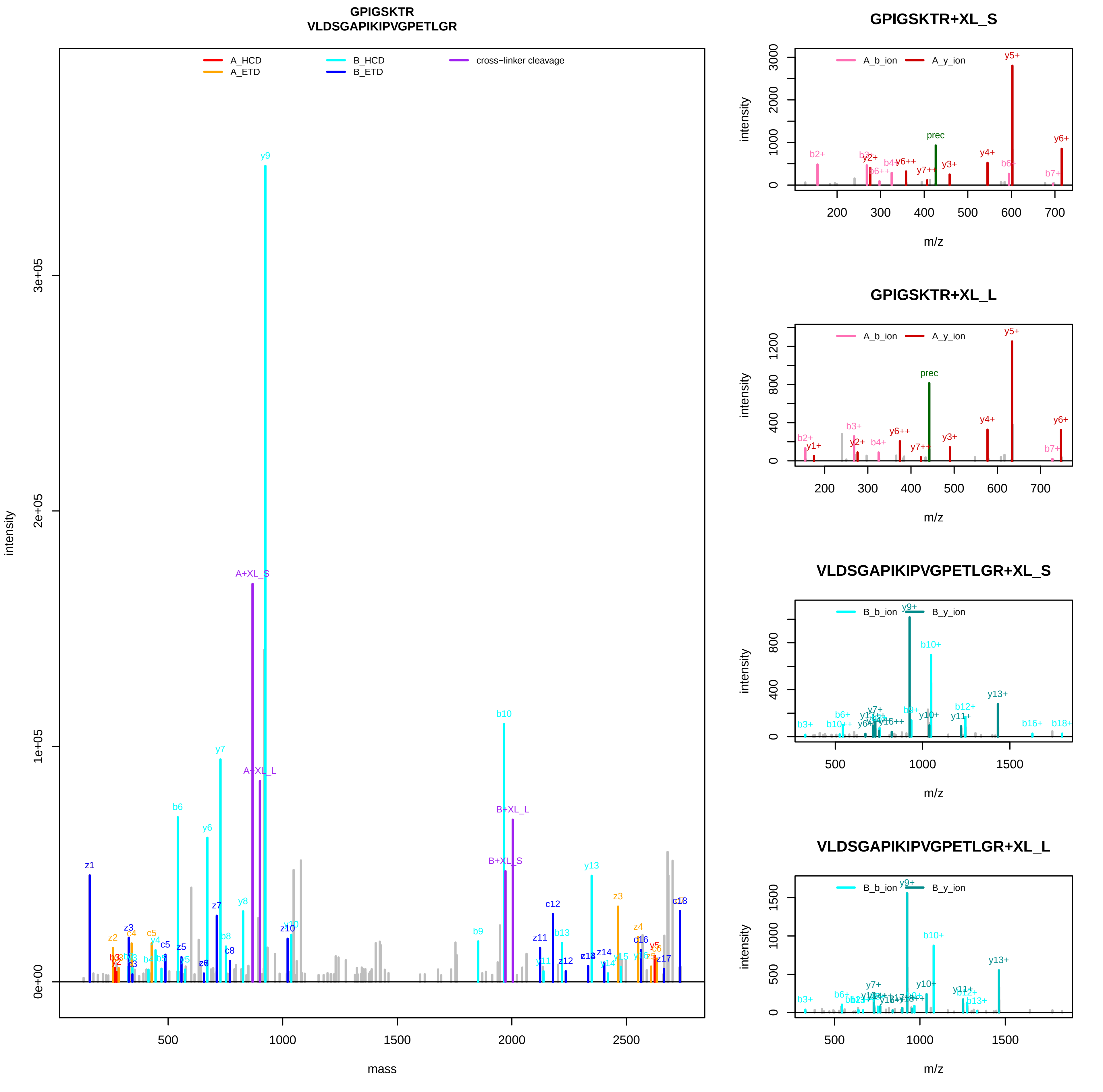

GPIGSKTR
VLDSGAPIKIPVGPETLGR
A_HCD
A_ETD
B_HCD
B_ETD
cross−linker cleavage
intensity
mass
0e+00
1e+05
2e+05
3e+05
500
1000
1500
2000
2500
y9
A+XL_S
A+XL_L
B+XL_L
B+XL_S
b10
y7
b6
y6
y13
z1
z3
z7
c12
z10
y8
z3
c18
b9
b13
z11
z4
c16
z12
z14
z17
GPIGSKTR+XL_S
A_b_ion
A_y_ion
prec
y5+
y6+
y4+
y3+
b2+
y2+
y6++
y7++
b6+
b7+
m/z
0
1000
2000
3000
200
300
400
500
600
700
GPIGSKTR+XL_L
y1+
b3+
b4+
0
400
800
1200
VLDSGAPIKIPVGPETLGR+XL_S
B_b_ion
B_y_ion
y9+
b10+
y13+
b12+
y11+
y10+
b16+
b18+
b3+
b6+
y7+
0
400
800
1000
1500
VLDSGAPIKIPVGPETLGR+XL_L
0
500
1000
1500
